# Alarmin S100A8/S100A9 as a biomarker for molecular imaging of local inflammatory activity

**DOI:** 10.1038/ncomms5593

**Published:** 2014-08-06

**Authors:** Thomas Vogl, Michel Eisenblätter, Tom Völler, Stefanie Zenker, Sven Hermann, Peter van Lent, Andreas Faust, Christiane Geyer, Beatrix Petersen, Kirsten Roebrock, Michael Schäfers, Christoph Bremer, Johannes Roth

**Affiliations:** 1 Institute of Immunology, University of Münster, 48149 Münster, Germany; 2 Interdisciplinary Centre for Clinical Research, University of Münster, 48149 Münster, Germany; 3 Division of Imaging Sciences and Biomedical Engineering, King’s College London, London SE1 7EH, UK; 4 Department of Clinical Radiology, University of Münster, 48149 Münster, Germany; 5 European Institute for Molecular Imaging, University of Münster, 48149 Münster, Germany; 6 Department of Rheumatology, Radboud University Medical Centre, 6500 HB Nijmegen, The Netherlands; 7 Cluster of Excellence EXC 1003 ‘Cells in Motion - CiM’, University of Münster, 48149 Münster, Germany; 8 Department of Radiology, St Franziskus Hospital Münster, 48145 Münster, Germany; 9 These authors contributed equally to this work

## Abstract

Inflammation has a key role in the pathogenesis of various human diseases. The early detection, localization and monitoring of inflammation are crucial for tailoring individual therapies. However, reliable biomarkers to detect local inflammatory activities and to predict disease outcome are still missing. Alarmins, which are locally released during cellular stress, are early amplifiers of inflammation. Here, using optical molecular imaging, we demonstrate that the alarmin S100A8/S100A9 serves as a sensitive local and systemic marker for the detection of even sub-clinical disease activity in inflammatory and immunological processes like irritative and allergic contact dermatitis. In a model of collagen-induced arthritis, we use S100A8/S100A9 imaging to predict the development of disease activity. Furthermore, S100A8/S100A9 can act as a very early and sensitive biomarker in experimental leishmaniasis for phagocyte activation linked to an effective Th1-response. In conclusion, the alarmin S100A8/S100A9 is a valuable and sensitive molecular target for novel imaging approaches to monitor clinically relevant inflammatory disorders on a molecular level.

## Abstract (web summary)

Alarmins are locally released during inflammation and are early amplifiers of inflammation. Here Vogl *et al.* show that the alarmin S100A8/S100A9 can be used as a sensitive marker to detect subclinical inflammation and follow disease progression in a variety of disease models.

## Graphical abstract

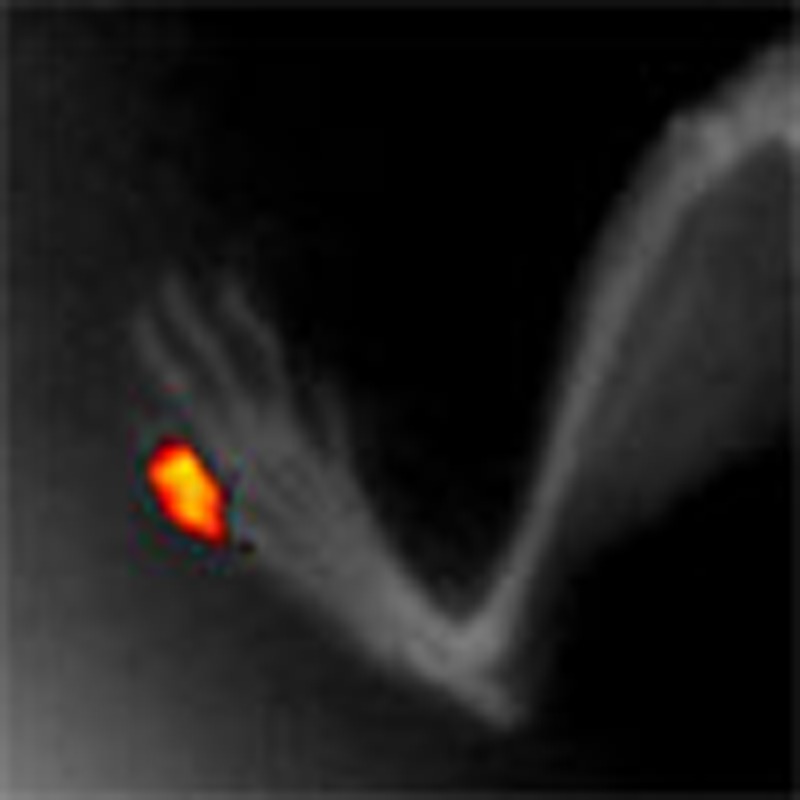

Inflammation is the driving force in a vast spectrum of clinically relevant disorders, among others recognized as a major pathological mechanism in malignant and degenerative diseases, infection and autoimmunity. Current imaging markers mostly reflect either metabolism or secondary effects of inflammatory reactions, such as increased perfusion or vessel permeability, or are only suitable for a very specific subset of diseases. In addition, all currently established biomarkers widely lack a proven prognostic potential. With biomedical research increasingly discovering the molecular and cellular basis of diseases and highly specific molecular therapies at the same time, both approaches do not provide sufficient diagnostic information. As a result, individually adapted therapy to manage chronic inflammatory diseases remains widely elusive despite significant therapeutic improvements^1^.

Numerous imaging approaches have been designed to address this issue. *In vivo* visualization of local inflammation has been performed, for example, using F-18-fluorodeoxyglucose (^18^F-FDG)-positron emission tomography (PET) or magnetic resonance imaging (MRI) with or without contrast enhancement^2^. Although these methods have proven diagnostic value, their implication in clinical practice has not fostered personalized therapy, mostly due to a lack of either desirable specificity (PET) or sensitivity (MRI).

Targeted imaging approaches to overcome these limitations would ideally address a biomarker with high expression/release or accumulation locally at the site of inflammation, representative of early inflammatory processes and residual disease activity or a prediction of flare-ups of disease in remitting-relapsing courses of chronic inflammation. In preclinical animal models, non-invasive molecular imaging methods would allow for local and longitudinal assessment of biomarkers in individual subjects. In the long-term, such biomarkers would facilitate individual adaptation of medication and would lead to a significant step forward in the concept of personalized medicine.

In recent years, the concept of alarmins or ‘danger-associated molecular pattern molecules’ (DAMPs) has emerged as a novel mechanism for initiating and promoting inflammation and has more recently been recognized as capable of resolving inflammation^3^,^4^,^5^,^6^. Expressed and released during tissue damage or cellular stress reactions, members of this protein family have been shown to be early players in the development of inflammatory processes. S100A8 and S100A9, two members of the DAMP-family, are highly expressed in early infiltrating phagocytes. During the activation of these cells, S100A8/S100A9 complexes are locally released in virtually all inflammatory disorders that are associated with phagocyte activation, like autoimmune diseases, rheumatoid arthritis, allergies, cardiovascular diseases, or local and systemic infections and tumours^7^, whereas virtually no expression can be found in healthy tissue. We have previously shown that S100A8 and S100A9 promote inflammation via the activation of Toll-like receptor-4 (refs 8, 9, 10, 11). Serum concentrations of S100A8/S100A9 complexes have been shown to be superior over conventional biomarkers for the monitoring of inflammatory disorders, especially in the detection of residual disease activity and in the prediction of relapse in arthritis^12^.

However, biomarkers measured in the blood only reflect the systemic state, which is strongly affected by factors like metabolism or blood clearance, limiting the specificity and sensitivity of these approaches. In contrast to systemic measurements, non-invasive imaging should be able to detect the expression of alarmins even at the local site of inflammation. Using fluorescence reflectance imaging (FRI), we now provide the first evidence that molecular imaging allows for the reliable detection of S100A8 and S100A9 in preclinical models, locally expressed during disease, and that visualization of these proteins in conjunction with further laboratory analysis enables the monitoring of local inflammation with unique sensitivity, even allowing for the detection of sub-clinical, residual disease activity. In autoimmune arthritis, we can simultaneously monitor multiple disease foci by *in vivo* S100A9 imaging and the extent of disease could be determined with high precision and even prognostic value for disease development in independent foci of the same animal. Moreover, by imaging S100A9 expression, we have demonstrated the first biomarker detecting subclinical differences in phagocyte activation linked to disease outcome in a model of Th1/Th2-dichotomy in response to leishmania infection. We provide evidence that S100A9 may serve as a novel potent biomarker for monitoring local inflammatory processes by molecular imaging. The broad applicability and consecutive potential impact on clinical practice is illustrated by assessing the performance of our integrated approach in exemplary models of innate and adaptive immunity, autoimmunity and infection, thus covering representative relevant pathomechanisms of inflammatory disorders.

## Results

### Cy5.5-coupled aS100A9 accumulates at sites of inflammation

**Figure 1:**
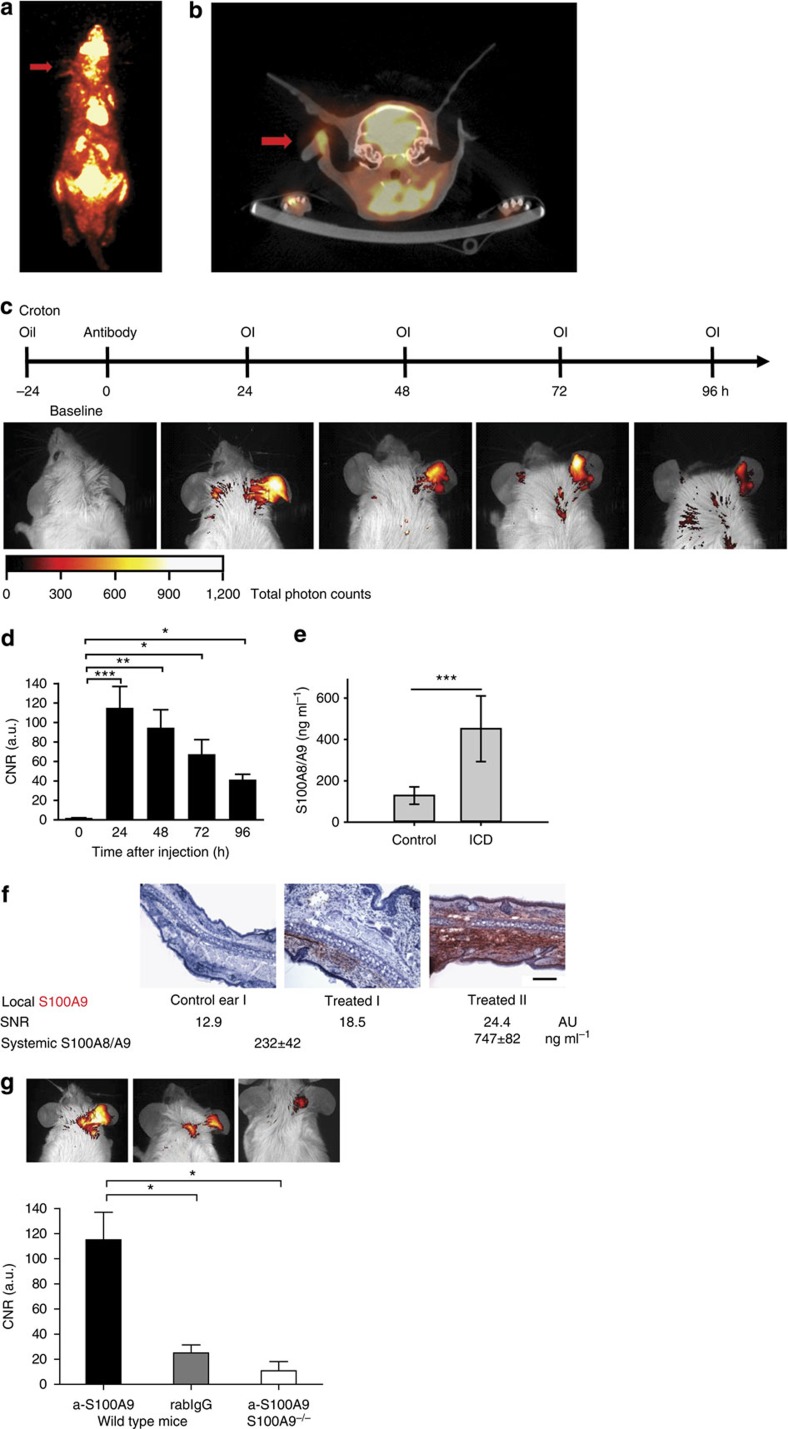
*In vivo* fluorescence reflectance imaging of mice during ICD. (**a**) ^18^F-FDG-PET image and the fused PET/CT (axial CT slice) (**b**) of ICD in mice treated with croton oil on the right ear. The area of inflammation (red arrows) can be depicted in the maximum intensity PET image (**a**, whole body) with the axial CT slice showing the swelling of the inflamed tissue and the local uptake of FDG (ratio inflamed ear versus healthy ear=4.8; **b**). (**c**) After the application of a-S100A9-Cy5.5 to Balb/c mice 24 h after elicitation of ICD, optical imaging (OI) was performed at the time points indicated. Strong fluorescence intensities were detected only at sites of inflammation for up to 96 h. (**d**) Quantification of CNR shows significant changes in the affected ears over the observed time period from 24 to 96 h (baseline=time point 0). Data are from three independent experiments (each *n*=5, mean±s.d., *P<0.05, **P<0.01, ***P<0.001; *P* values calculated using Student’s *t*-test). (**e**) S100A8/S100A9 serum concentrations 48 h after croton oil application. Data are from five mice per group (mean±s.d., ***P<0.001; Student’s *t*-test). (**f**) Cryosections of treated and control ears were stained for S100A9-expression. The figure shows representative ear sections of an untreated control ear (left) and treated ears with moderately (middle) and strongly (right) elevated SNR including the corresponding systemic S100A8/S100A9 level. Scale bar, 100 μm. (**g**) Application of a-S100A9-Cy5.5 or rabIgG-Cy5.5 to WT or S100A9^−/−^ mice 24 h after the elicitation of ICD confirmed the specificity of optical imaging for S100A9 expression *in vivo*. Data are from five mice per group (mean±s.d., *P<0.05; *P*-values calculated using one-way analysis of variance with Bonferroni’s post test). a.u., arbitrary units.

To evaluate the feasibility of monitoring S100A9 expression by optical imaging, we employed irritant contact dermatitis (ICD) as an inflammatory model, exclusively driven by innate immunity, independent of the adaptive immune system. ICD was induced in Balb/c mice by the local application of croton oil towards the ear skin^13^. ^18^F-FDG-PET, as a clinically established imaging technique to assess inflammatory activity^14^, was used to visualize elevated glucose metabolism in areas of inflammation during ICD. Although inflammation can be clearly depicted by the maximum intensity PET image (Fig. 1a, red arrow), as well as in the co-registered PET/ computed tomography (CT; Fig. 1b, axial CT slice), the resulting contrast-to-noise ratio (CNR) is low due to ^18^F-FDG being taken up by all glucose-consuming cells (Fig. 1a).

Cy5.5-labelled antibodies against S100A9 (a-S100A9-Cy5.5, 2 nmol of dye/mouse, spectral characteristics: 
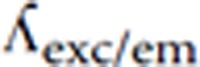
=680/700 nm, band pass=20 nm) were administered intravenously 24 h after croton oil application. Optical imaging was performed repeatedly 24–96 h after tracer application. Local tracer accumulation, as depicted by FRI, showed the highest level at 24 h (Fig. 1c,d). Fluorescence signals reflecting the high local release of S100A8/S100A9 correlated well with elevated systemic levels of S100A8/S100A9 complex in the serum of mice 48 h after croton oil application, as quantified by ELISA (Fig. 1e, Supplementary Fig. 1). Local S100A9 expression could be confirmed using immunohistochemistry (Fig. 1f). In healthy animals, a-S100A9-Cy5.5 showed a biodistribution that is typical for macromolecular substances (Supplementary Fig. 2). To discriminate nonspecific tracer distribution, rabbit IgG of irrelevant specificity was labelled with Cy5.5 (rabIgG-Cy5.5) and injected into a control group of mice. S100A9-deficient mice (S100A9^−/−^) served as additional controls for the specificity of tracer to target binding. Both sets of controls showed only a slight increase in fluorescence at local sites of inflammation, most likely reflecting hyperemia and F_c_γ receptor expression/binding in areas of inflammation (Fig. 1g). The distribution of rabIgG-Cy5.5 and a-S100A9-Cy5.5 in either healthy wild-type (WT) animals or non-involved organs was virtually identical (Supplementary Fig. 2).

### Monitoring local inflammation in allergic contact dermatitis (ACD)

**Figure 2:**
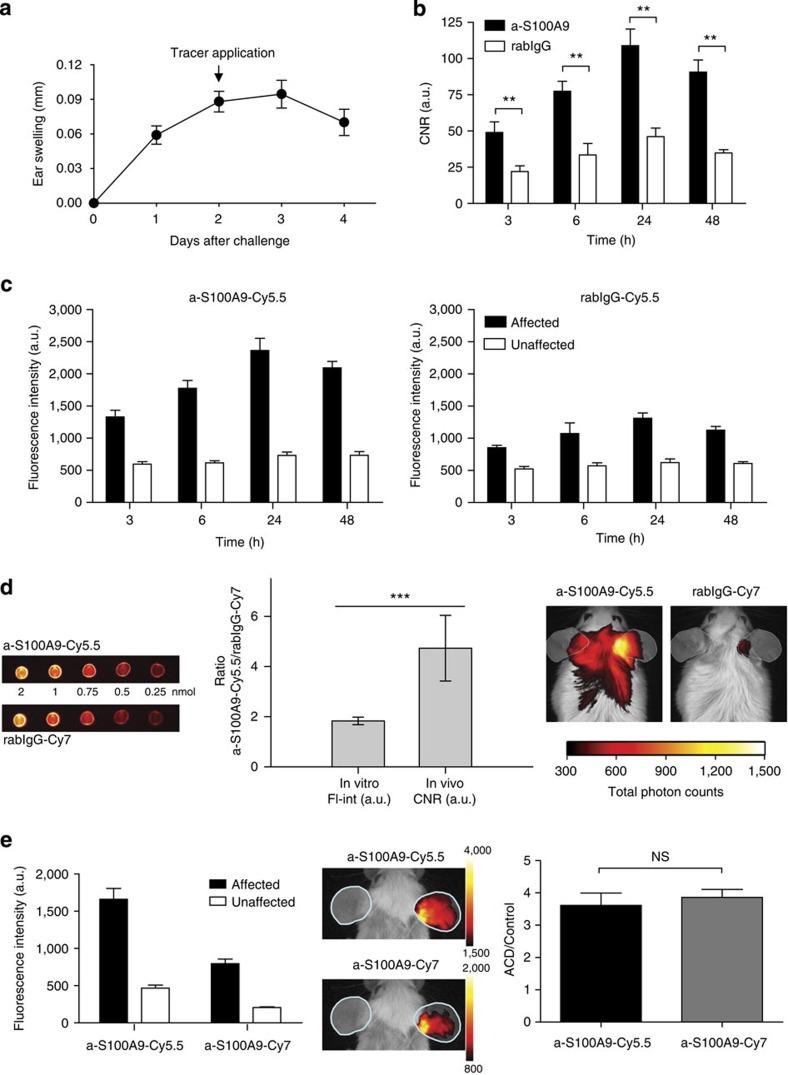
Monitoring of S100A9 expression during ACD by fluorescence reflectance imaging (FRI) *in vivo.* (**a**) ACD was induced in mice and disease progression was assessed by the increase in ear swelling. (**b**) FRI was performed at different time points after the application of either a-S100A9-Cy5.5 or rabIgG-Cy5.5, as indicated in the figure. The optimal time point for optical imaging was found to be 24 h after tracer application. (**c**) Fluorescence intensities of the specific (left image) versus unspecific tracer (right image) of affected (black bars) versus unaffected (white bars) ears allowed for the estimation of F_c_γ receptor contribution to total FRI signals. (**d**) Comparison of the ratios of equal amounts of a-S100A9-Cy5.5 and rabIgG-Cy7 *in vitro* (FL-int=fluorescence intensities, left side) versus *in vivo* (CNR, right side). ACD was induced in mice and 2 nmol of Cy5.5-labelled anti-S100A9 and Cy7-labelled rabIgG were injected simultaneously. Optical imaging was performed 24 h after antibody injection and the region of interest (ROI) of data acquisition was labelled in cyan. Data are from five mice per group (two independent experiments each, mean±s.d.) *P<0.05, **P<0.01, ***P<0.001; Student’s *t*-test. (**e**) Comparison of the ratios of equal amounts of a-S100A9-Cy5.5 and a-S100A9-Cy7 during ACD. ACD was induced in mice and 2 nmol of Cy5.5- and Cy7-labelled anti-S100A9 each was injected simultaneously. Optical imaging was performed at 24 h after antibody injection and the ROI of data acquisition was labelled in cyan (data are from five mice, mean±s.d. according to Student’s *t*-test). a.u., arbitrary units; NS, not significant.

To assess the capability of S100A9 imaging to reflect phagocyte activity in T-cell-dependent adaptive immune reactions, we analysed the expression of S100A9 in a model of ACD^13^. Two days after the allergen challenge of sensitized mice, a-S100A9-Cy5.5 or rabIgG-Cy5.5 was administered and fluorescence intensities were monitored for up to 48 h after tracer application. Disease progression was assessed by monitoring the ear swelling during ACD (Fig. 2a). Already after 3 h, the specific probe accumulated in the area of inflammation, peaking at 24 h (Fig. 2b). FRI data (Fig. 2b,c) were again in good accordance with increased S100A8/S100A9 serum levels of 258±116 ng ml^−1^ at day 3 (*P*<0.01) and of 289±91 ng ml^−1^ at day 4 (*P*<0.001) compared with control mice (129±42 ng ml^−1^, five mice per group (two independent experiments, mean±s.d)) and clinical symptoms (ear swelling; Fig. 2a). Similar fluorescence intensities of specific and nonspecific antibodies were obtained in unaffected ears, representing a perfusion background signal (Fig. 2c). To further differentiate the accumulation of labelled specific versus unspecific antibody in the region of inflammation, 2 nmol of both Cy5.5-labelled anti-S100A9 and Cy7-labelled rabIgG were injected simultaneously *in vivo* during ACD. Fluorescence signals obtained after the separate excitation of both probes were acquired and compared with data derived *in vitro* under defined conditions. The Cy5.5/Cy7 (reflecting a-S100A9/rabIgG) ratio *in vitro* was constant (1.83±0.15); however, we observed a significant increase to 4.73±1.31 *in vivo* (Fig. 2d). To rule out the effects of the dye properties on probe distribution, a-S100A9-Cy5.5 and a-S100A9-Cy7 were used simultaneously. An identical biodistribution of the differently labelled probes could be observed. Although a direct comparison of absolute signal intensities was obviated by different emission wavelengths and different quantum yields, the signal ratios between the affected and unaffected ear were virtually identical (Fig. 2e). A parallel injection of a-S100A9-Cy5.5 and rabIgG-Cy5.5 did not result in a further increase in the local signal compared with the injection of a-S100A9-Cy5.5 alone. As the S100A9-specific probe is based on a polyclonal antibody and thus presumably contains only a relatively small fraction of antigen-specific antibodies, these data confirm the specificity of our findings (Supplementary Fig. 3a). Immunohistochemistry showed a significant infiltration by CD11b myeloid cells, Gr-1 granulocytes and F4/80 macrophages as a source of locally released S100A8/S100A9 (Supplementary Fig. 3b).

### Monitoring local activity of inflammation in arthritis

S100A8 and S100A9 are highly expressed in synovial tissue in rheumatoid arthritis and serum concentrations have been shown to be reliable biomarkers reflecting local disease activity^10^,^11^,^15^. We performed optical imaging of S100A8 and S100A9 expression *in vivo* in a murine collagen-induced arthritis (CIA) model and correlated imaging data with the clinical scores of disease activity. CIA was induced by immunization of DBA/jdba1/j mice with type II collagen. Clinical manifestations of arthritis started around day 7 after the last collagen injection. Symptoms prevailed for up to 3 weeks to different degrees in the foot joints. Mice received labelled antibodies 1 week after the last collagen injection (day 28). Optical imaging was performed starting 24 h after dye application.

**Figure 3:**
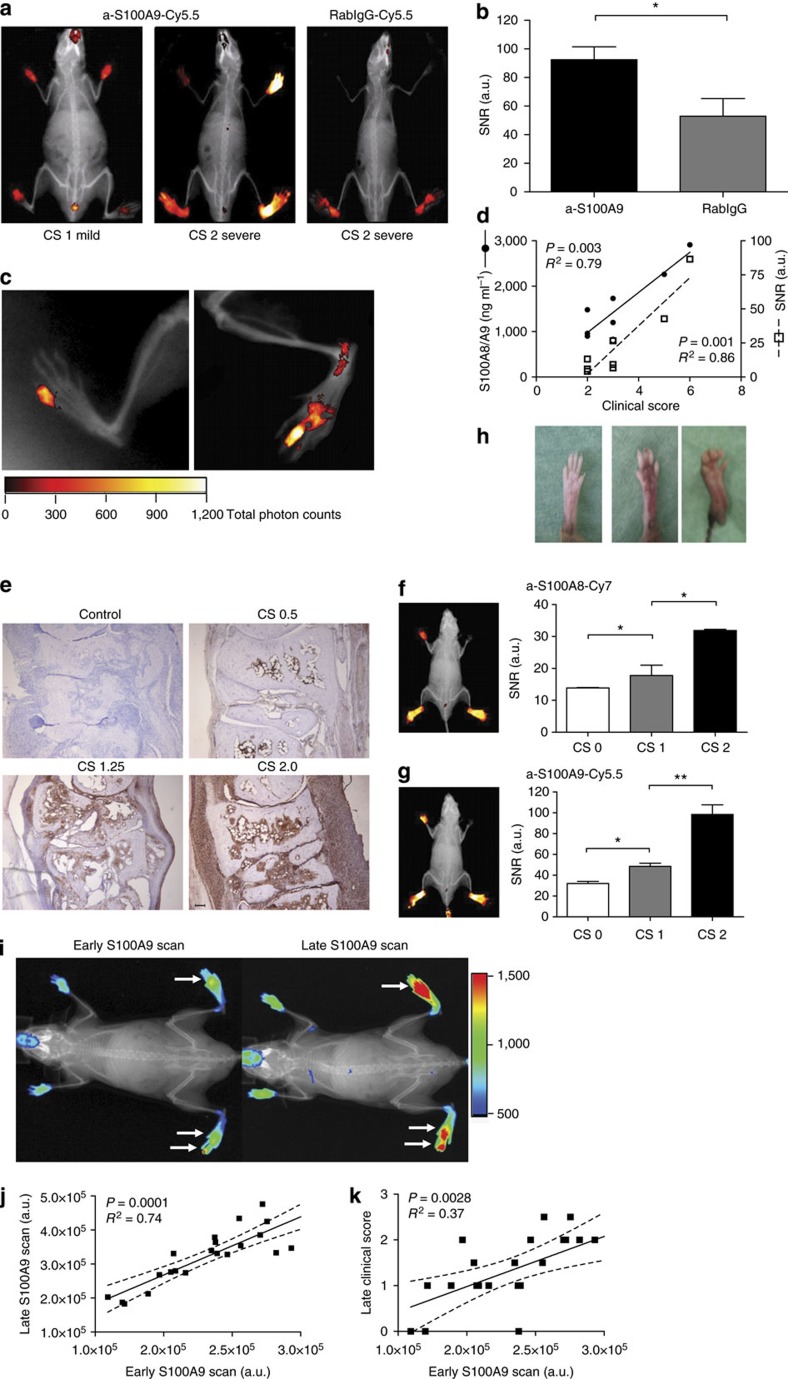
Detection of single inflamed joints in collagen-induced arthritis by optical imaging. (**a**) CIA was induced in DBA/jdba1/j mice and optical imaging was recorded by FRI using a-S100A9-Cy5.5 or rabIgG-Cy5.5 at day 28. Individual feet were analysed and compared with clinical scores described in the Method section (*n*=5 mice per group, three independent experiments). (**b**) Comparison of imaging data of a-S100A9-Cy5.5 and rabIgG-Cy5.5 from mice feet with CS2 confirms the specificity of our findings (mean±s.d., *P<0.05, Student’s *t*-test, *n*=5 mice per group, three independent experiments). (**c**) Enlarged view of only subclinical inflammation of single joints. (**d**) Correlation of imaging data and systemic S100A8/S100A9 levels of mice with clinical disease severity. Scoring of single feet were added up (maximum score 8 per mouse) and imaging data were calculated as mean values over all four feet. (**e**) S100A9 immunostaining of paw sections with CS of 0.5, 1.25 and 2 and control at day 28 confirmed correlation of local S100A8/S100A9 expression and severity of inflammation. Scale bar, 100 μm. (**f**,**g**) Simultaneous injection of a-S100A8-Cy7 (**f**) and a-S100A9-Cy5.5 (**g**) shows an almost identical distribution pattern in FRI. (**h**) Upper panel, representative pictures of paws for the different clinical scores. Lower panels, correlation of clinical scores with SNR for Cy7-labelled anti-S100A8 and Cy5.5-labelled anti-S100A9 (*n*=5 mice, mean±s.d., *P<0.05, **P<0.01; one-way analysis of variance with Bonferroni’s post test). (**i**) CIA was induced in C57BL/6 mice and a-S100A9-Cy5.5-driven FRI was performed at the first signs of arthritis (early time point) and for a second time where disease progression had occurred (late time point, 2 nmol of dye per mouse). All paws were analysed separately and compared with the clinical scoring as described in (**a**, *n*=6 mice). Representative images of an early (left image) versus late (right image) S100A9 scan are shown. White arrows indicate inflamed areas. Correlation of optical imaging data of early versus late time point (**j**) and early time point versus late clinical score (**k**). To correct for the variable areas of fore- and hind-paws, fluorescence intensities were normalized for the ROI size. Two fore-paws of one mouse were excluded from the analysis because of an incorrect position in the scanner (**j**,**k**). a.u., arbitrary units.

Owing to the highly variable inflammatory response of individual joints in CIA, we performed a clinical scoring for each individual foot on the basis of a three-point scale (CS0, CS1 and CS2) accounting for redness, swelling and deformation. S100A9 expression, as depicted by optical imaging, showed excellent correlation with clinical scoring, clearly discriminating clinically mild from severe joint inflammation (Fig. 3a) with a high signal-to-noise ratio (SNR) for severely inflamed joints (Fig. 3b). Even single affected small joints could be clearly identified (Fig. 3c). The cumulative disease activity score of all four feet (range 0–8) correlated well with optical imaging data presented as mean values for all four feet and systemic S100A8/S100A9 levels of 1,180±360 ng ml^−1^ in mice with mild arthritis (CS2-3) versus 170±30 ng ml^−1^ in healthy control mice and 2,700±380 ng ml^−1^ in mice with high disease activity (CS4-6; Fig. 3d,e). To assess unspecific tracer distribution, we employed non-targeted Cy5.5-labelled rabIgG and observed only faint tracer signals, even in strongly inflamed joints (Fig. 3a). The simultaneous injection of Cy7-labelled antibodies against the S100A8 subunit of the S100A8/S100A9 heterodimer (a-S100A8-Cy7, 2 nmol dye/mouse, spectral characteristics: _exc/em_=755/780 nm, band pass=20 nm) and a-S100A9-Cy5.5 showed an almost identical *in vivo* distribution with equally specific accumulation in target areas (Fig. 3f,g). SNR of a-S100A8-Cy7 and a-S100A9-Cy5.5 showed excellent correlation with disease activity scores of individual feet (Fig. 3h). To further assess a potential prognostic value of the presented approach, we conducted the CIA model in C57BL/6 mice. Development of arthritis and outcome in this mouse strain is highly variable and unpredictable. Even during early disease, when first clinical signs of CIA could only just be detected, scans allowed for the safe delineation of areas of S100A9 expression. All four paws were scored daily and imaging was repeated when manifesting clinical signs of inflammation were present. We also demonstrated an excellent correlation between early and late imaging (Fig. 3i,j), as well as between early imaging and the clinical development of individual paws (Fig. 3k).

### Phagocyte activity during *Leishmania major* infection

Experimental *leishmaniasis* is a well-established model for analysing the Th1/Th2-dependent immune response to infection. C57BL/6 mice show early activation of phagocytes within the first days after *Leishmania major (L. major)* inoculation, which results in an effective Th1-response and the clearance of parasites. In contrast, Balb/c mice lack the sufficient activation of phagocytes in the initial phase of infection, finally resulting in the development of a Th2-dominated, ineffective immune response and subsequent parasite dissemination. The consecutive granuloma formation is accompanied by an overwhelming yet inefficient phagocyte activation in the late phase of disease^16^.

**Figure 4:**
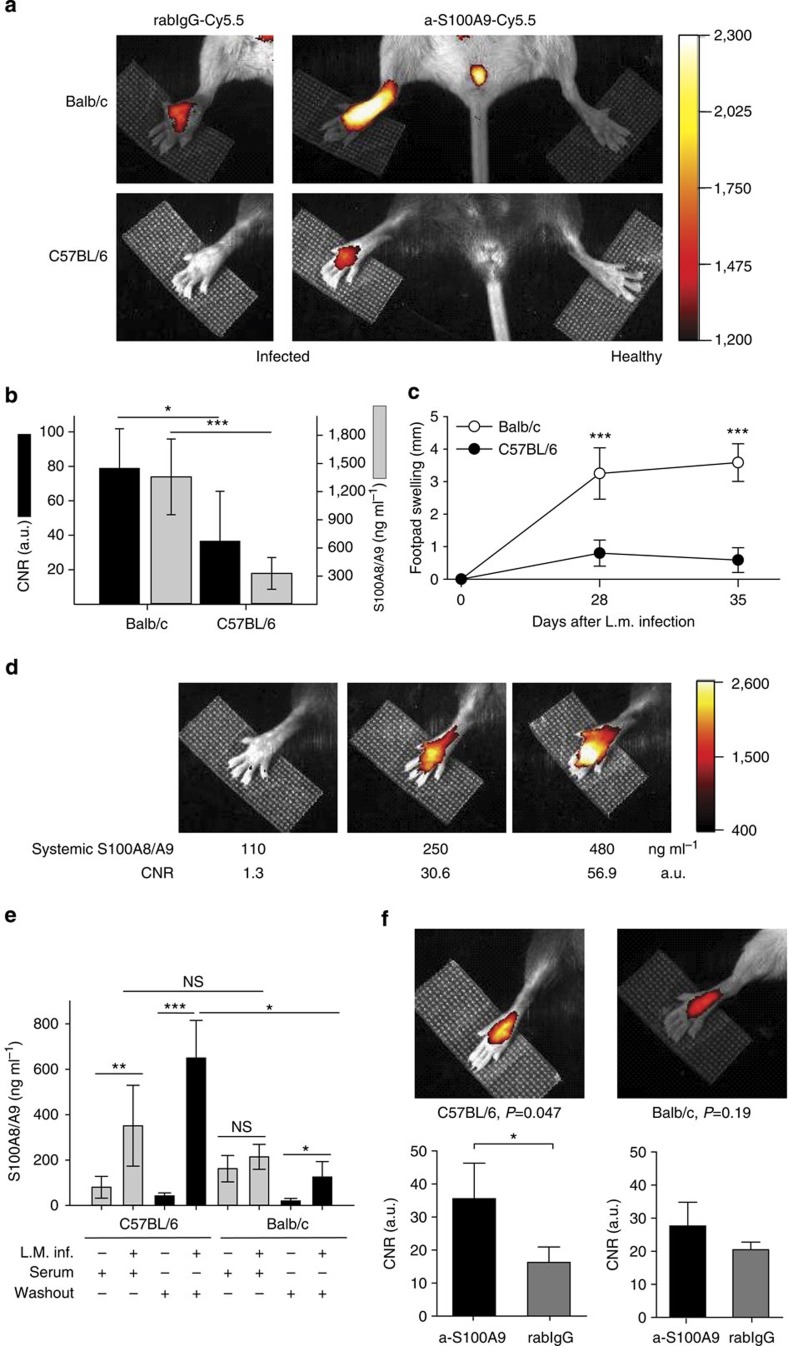
Mouse strain-specific responses during *L. major* infection monitored by S100A9 imaging *in vivo.* (**a**) Right hind legs of C57BL/6 mice and Balb/c mice (three independent experiments, each five mice per group) were infected with *L. major,* whereas the left hind legs served as controls. FRI was monitored during the late phase of infection at day 28 after receiving either a-S100A9-Cy5.5 or rabIgG-Cy5.5 (2 nmol of dye per mouse) 24 h earlier. (**b**) CNR was calculated for both mouse strains at day 28 after *L. major* infection. Significant strain-specific differences were found for both local (CNR) and systemic (S100A8/S100A9) parameters (three independent experiments, each five mice per group, mean±s.d., *P<0.05, ***P<0.001; Mann–Whitney *U*-test). (**c**) Footpad swelling of infected C57BL/6 mice and Balb/c mice at day 28 and 35 in relation to non-infected contralateral foot pads (mean±s.d., *n*=5 for each mouse strain, ***P<0.001; *t*-test) demonstrates the different outcome in both mouse strains. (**d**) Individual comparison of representative optical imaging data (CNR) of infected C57BL/6 mice at day 28 after infection shows fairly good accordance, suggesting that systemic S100A8/S100A9 levels resemble disease activities. (**e**) During early *L. major* infection (day 4), sera (grey bars) and footpad washouts (black bars) of infected and non-infected mice were collected and analysed for S100A8/S100A9 by ELISA. Systemic and local S100A8/S100A9 levels were already significantly increased in infected C57BL/6 mice as compared with controls. In Balb/c mice, only a minor, nonsignificant increase in local S100A8/S100A9 expression was observed. Data are from fvie mice per group (mean±s.d., *P<0.05, **P<0.01, ***P<0.001 and NS, not significant; Mann–Whitney *U*-test). (**f**) At day 4 already, local upregulation of S100A9 expression could be monitored in resistant C57BL/6 mice by optical imaging (*P*=0.047 by *t*-test) reflecting phagocyte activation. This was not detectable in susceptible Balb/c mice (*P*=0.19). Data are from five mice per group. a.u., arbitrary units.

At day 28 after infection, in the late phase of disease, optical imaging of infected animals was performed, showing significantly higher fluorescence signals in the feet of infected Balb/c mice as compared with C57BL/6 mice and untreated control feet (Fig. 4a, right images). RabIgG-Cy5.5 confirmed the specificity of S100A9-labelling (Fig. 4a, left images). We observed an excellent correlation of optical signals with systemic S100A8/S100A9 levels (Fig. 4b) and footpad swelling (Fig. 4c) as a clinical sign of inflammation. Moreover, in C57BL/6 mice, even at day 28 after infection, fluorescence-reflected macrophage activity allowed the grading of residual inflammation (Fig. 4d).

Using S100A9 imaging, even the critical, initial activation of the phagocyte system in *L. major*-resistant C57BL/6 mice could be detected during the very early phase at day four after infection. Measurements of S100A8/S100A9 expression in sera or local wash-outs by ELISA confirmed the detected imaging signals (Fig. 4e). In contrast, the failure of sufficient early phagocyte activation in Balb/c mice was associated with the absence of a significant increase in S100A9 expression (Fig. 4f). We could therefore demonstrate that locally expressed S100A9 is the first imaging marker predicting the development of a Th1 immune response, several weeks before the clinical outcome of infection.

## Discussion

Inflammatory disorders like autoimmune diseases, allergies and acute or chronic infections are important challenges in health care. The continuously growing understanding of the biological basis of inflammation stimulates the development of targeted therapies, specifically addressing selected steps in the process of pathogenesis. This is accompanied by a growing demand for specific diagnostic approaches, which are capable not only of sensitive detection but also of characterization of the disease stages on a molecular level. Molecular biomarkers that meet the requirements as set out by current therapeutic developments—sensitive reflection of disease activity changes under therapy, safe visualization of subclinical disease activity as a sign of therapy failure and a prediction of the outcome—are still missing. However, these requirements are a prerequisite for the adaptation of personalized therapeutic approaches providing optimal therapy.

Analysing S100A8 and S100A9, two members of the DAMP family, our study comprises a novel diagnostic approach: DAMPs or alarmins are widely accepted to represent initial tissue signals in response to cell stress and tissue damage, thus representing ideal candidates for the early and sensitive detection of developing inflammation^3^,^5^,^17^. Both proteins are among the most upregulated genes in numerous inflammatory diseases^7^,^15^. S100A8/S100A9 complexes are secreted during the activation of immigrating phagocytes or released by necrotic cells because of tissue damage at local sites of inflammation, acting as endogenous triggers of Toll-like receptor-4 and inducing the expression of pro-inflammatory signalling molecules in phagocytes, lymphocytes, endothelial and epithelial cells and osteoclasts^8^,^10^,^18^,^19^. In this context, S100A8 and S100A9 show typical characteristics of alarmins or DAMPs. Targeting S100A8/S100A9, we therefore monitored an inflammatory mechanism that has been shown as highly relevant in different mouse models of inflammation like allergies, autoimmune diseases, arthritis and infection and has also proven to be a valuable marker for discrimination and grading of clinically relevant acute and chronic inflammation^7^,^8^,^9^,^10^,^13^,^18^,^19^.

Targeted imaging of cytokines, for example, interleukin-1β or tumour necrosis factor-α revealed only limited sensitivity and/or specificity and a relatively low SNR^20^,^21^,^22^, probably due to a low gradient of the cytokine concentration between local inflammatory exudates and systemic serum. In contrast to most cytokines, chemokines and other pro-inflammatory molecules, the local accumulation of S100A8 and S100A9 is very high (up to 100 μg ml^−1^ and about 50- to 100-fold higher than systemic concentrations), allowing the sensitive and specific detection of local changes in the expression of these molecules^15^,^23^. S100A8 and S100A9 comprise about 40% and 5% of the cytosolic proteins in granulocytes and monocytes, respectively. They are specifically released during the interaction of phagocytes with inflammatory activated endothelial cells and can subsequently bind to heparan sulfate of the endothelial glycocalyx^15^,^24^,^25^,^26^. The high abundance and matrix binding appear to be responsible for the high local accumulation of these proteins at sites of inflammation compared with other inflammatory molecules, which allows the detection of these molecules even by MRI^27^ and accounts for the high target to background ratios that we observed in our *in vivo* imaging studies.

In contact dermatitis, as a model of local inflammation, we could demonstrate that the expression of S100A9 is a very sensitive marker for inflammatory processes and reflects disease activity independently of the underlying pathomechanism, for example, in toxic or in allergen-induced inflammation. In contrast to most imaging studies, we confirmed that our imaging data closely correlated not only to the local expression of S100 proteins by phagocytes in the dermal infiltrate, as determined by immunohistochemistry, but also to systemic levels of both proteins and clinical parameters. The specificity of our data was confirmed by the use of nonspecific antibodies (rabbit-derived IgG without relevant specificity) to determine perfusion and unspecific F_c_γ-receptor binding. Parallel injection of the S100A9-specific probe and rabIgG labelled with different dyes ruled out *in vivo* competition for the specific target or dye-dependent effects. Moreover, we applied the specific probe a-S100A9-Cy5.5 in ICD in WT and S100A9^−/−^ mice. SNR of a-S100A9-Cy5.5 in WT mice were significantly higher compared with nonspecific antibodies, as well as compared with data obtained from S100A9^−/−^ mice. In this model, WT and S100A9^−/−^ mice showed no differences regarding their inflammatory phenotype^13^.

Analysing CIA, we confirmed an excellent correlation of S100A9 expression, as detected by optical imaging with local disease activity in individual joints. In addition, we demonstrated that the local expression of S100A9 and S100A8 is equally capable of uncovering sub-clinical disease activity, which is not reflected by established clinical parameters. Using blocking antibodies in arthritis models in WT and S100A9^−/−^ mice revealed that this alarmin has a pivotal role in the inflammatory as well as in the destructive process during arthritis^10^,^11^,^28^, indicating that our imaging approach directly reflects major pathogenic aspects of arthritis.

In CIA, E-selectin-targeted optical imaging has been demonstrated to reflect inflammatory activity with similar SNR to our data^29^. Interestingly, S100A9 has been shown to induce the expression of E-selectin in endothelial cells^30^, indicating that both methods address different target cells within closely linked inflammatory mechanisms. However, the high local level of S100A9 at sites of numerous inflammatory diseases makes this molecule more suitable for future imaging approaches in clinical practice. The detection of leukocyte populations with radio-labelled antibodies against macrophages (F4/80), T lymphocytes (CD4, CD3, CD40) or B lymphocytes (CD20), among others, and cell tracking studies with labelled cells have been used to monitor inflammatory processes in arthritis^31^,^32^. However, all of these approaches suffer from the disadvantage that the infiltration of significant numbers of these leukocytes takes a substantial amount of time and is not the initial event in the inflammation cascade. Moreover, leukocytes may persist during the resolution of inflammation, limiting the diagnostic value of these approaches for monitoring relapsing remitting courses of inflammation. Therefore, most of these studies have identified only low SNR and sometimes entirely failed to show a correlation of imaging results with disease activity^31^,^32^. The tissue destruction associated with phagocyte infiltration is reflected by protease activity and can be detected by small compounds targeting myeloperoxidase, cathepsin or matrix metalloproteinase activity^33^,^34^,^35^. However, these markers, in contrast to S100A8/S100A9, are not sensitive for the early inflammatory reaction, but reflect later stages of disease only.

Yet another interesting finding in our study was that the expression of S100A8/S100A9 during the initial phase of an inflammatory (infectious) process was the first early and sensitive marker for subclinical phagocyte activation known to be linked to the development of an effective Th1-response in this model. During the very early phase of *L. major* infection (day 4), we were able to visualize the effective activation of phagocytes in resistant C57BL/6 mice. In susceptible Balb/c mice, the lack of adequate, early phagocyte activation and differentiation into a pro-inflammatory M1-phenotype was reflected by low S100 expression in our imaging approach associated with priming a Th2 response, not sufficient to fight the infection. Hence, the imaging signal allowed for the prediction of a fatal outcome of disease, even weeks before full manifestation of the clinical phenotype and disease dissemination in this particular disease model.

Our data demonstrate for the first time that targeting members of the alarmin or DAMP family is a potent strategy for functional molecular imaging of inflammatory processes in general and independent of the underlying pathophysiology. Similar results of local expression of an alarmin family member could be demonstrated using a transgene reporter mice model for HSP-70 expression in ischemic brain injury. However, such transgenic approaches unfortunately only have limited potential for translation into clinical applications^36^. Owing to their specific mode of expression and release, S100A8 and S100A9 are sensitive biomarkers for the immediate response of innate immune mechanisms to disturbances of tissue homeostasis. The induction and release of S100A8/S100A9 have been shown to correlate very well with disease activity in many clinically relevant disorders, including rheumatoid arthritis, inflammatory bowel disease, autoimmune diseases, infections, allograft rejection or chronic processes like atherosclerosis, which underlines the translational potential and high impact of our findings for future basic research as well as clinical applications^3^,^7^,^12^. Optical imaging driven by fluorescently labelled antibodies has a growing translational potential beyond its impact on preclinical research and may be applicable for the examination of superficial lesions such as cutaneous inflammation. In addition, fluorescence endoscopy is gaining increasing interest and could foster the clinical use of fluorescent contrast agents^37^; for example, in the evaluation of inflammatory bowel disease, which is known to exhibit a very high local expression of S100A8/S100A9 (calprotectin)^38^. The potential of optical imaging of S100A8/S100A9 for regular clinical use is moreover underlined by the introduction of an optical scanner for visualization of the disease activity in rheumatoid arthritis, another inflammatory disorder with high local S100A8/S100A9 expression, currently driven by ICG fluorescence^39^. Finally, S100A8/S100A9 are highly upregulated during tumour development and even in the developing metastatic niche^40^. Therefore, another specific demand for optical molecular imaging of S100 proteins may arise in the context of intraoperative imaging for the safe delineation of malignant tissue during tumour resection^41^.

For the translation of target-specific imaging approach into deep tissue imaging, other label strategies may have to be explored. Replacing the fluorescent dye with a radionuclide for either single photon emission computed tomography (SPECT) or PET imaging would allow for the examination of virtually all body compartments in humans, with specificity and sensitivity resembling that of optical imaging. With hybrid systems like PET/CT and PET/MRI increasingly applied in clinical imaging, targeted imaging of S100A8/A9 could be combined with high-resolution morphology. Targeted contrast agents for MRI, based on either superparamagnetic iron oxides or Gadolinium, suffer from the low sensitivity of MRI at clinical field strength and—in comparison to optical or radionuclide-driven imaging—large amounts of the agent are required to incur a measurable change in relaxivity^42^. Although the visualization of tracer accumulation in areas of inflammation could be demonstrated for Gadolinium-loaded nanoparticles in selected experimental models^27^, a convincing approach with translational potential has yet to be developed. Moreover, the combinatory application of contrast enhanced MRI and targeted imaging in hybrid systems to acquire even more diagnostic information in a single examination would be negated by the use of targeted MRI probes. As a paradigm for such future developments in molecular imaging, we provide the first fully integrated diagnostic approach on a member of the DAMP/alarmin family in various preclinical models of different modes of inflammation with clear potential for translation into clinical practice.

## Materials and methods

### Mice and reagents

C57BL/6 mice, Balb/c mice (Harlan Laboratories), DBA/jdba1/j mice (Janvier-Elevage) and S100A9-deficient mice (S100A9^−/−^, backcrossed to C57BL/6 or Balb/c background (F10 generation))^43^ were used at the age of 8–12 weeks, sex matched for each set of experiments and housed under specific pathogen-free conditions. All experiments with mice were performed with the approval of the State Review Board of Nordrhein-Westfalen (Germany) according to the German law for animal welfare (Permit Number: 84-02.04.2012.A058) or by the Ethics Committee of University Hospital Nijmegen (Permit Number: DEC 2014-044). All reagents were purchased from Sigma at the highest purity grade available, unless indicated otherwise.

### ELISA

We used an in-house ELISA to determine the concentrations of S100A8/S100A9 in sera and washouts of footpads, as described earlier (Supplementary Fig. 1)^10^. We calibrated our ELISA against purified S100A8/S100A9 heterodimer as complexes have been shown to be the predominant form of these proteins.

### Antibodies and antibody labelling

Rabbit-derived antibodies addressing S100A9 or S100A8 were purified via protein G-sepharose and labelled with the fluorochromes Cy5.5 or Cy7 according to the manufacturer’s instructions (GE Healthcare). Cy5.5-labelled rabbit IgG without relevant specificity in mice served as a control. Briefly, 5 mg of the antibody was dialysed towards 100 mM Na_2_CO_3_ buffer, pH 8.0 and a 20-fold excess of the fluorochrome was added for 90 min at RT. The resulting tracer was purified from unbound dye using size exclusion chromatography (PD10 column). The labelling efficacy (dye/antibody ratio) was determined on the basis of ultraviolet-spectra of the purified dye–antibody compound using PBS as a reference buffer. Typically, the labelling resulted in 2.5–3.0 fluorochrome molecules per antibody, irrespective of the precursors.

### *In vivo* imaging

Mice were held under isoflurane inhalation anaesthesia for the duration of the scan and the imaging chamber was heated to 30°. As the total scan time was usually under 1 min, physiological effects due to a significant decrease of body temperature were not to be expected. Mice were intravenously injected with either the specific Cy5.5-labelled S100A9 antibody (a-S100A9-Cy5.5, 2 nmol of Cy5.5 ~100 μg antibody in total) or Cy5.5-labelled antibody of irrelevant specificity (rabIgG-Cy5.5), unless specified otherwise. In selected experiments, a-S100A9-Cy5.5- and Cy7-labelled S100A8 antibodies (a-S100A8-Cy7) or a-S100A9-Cy5.5- and Cy7-labelled S100A9 antibodies (a-S100A9-Cy7) or a-S100A9-Cy5.5 and rabIgG-Cy5.5 antibodies were administered in parallel in the same animal for simultaneous detection.

### FRI

FRI was performed using the Carestream FX Pro Imaging Station (Carestream Health). For imaging of Cy5.5-labelled antibodies, excitation light was set to 630 nm using an appropriate bandpass filtre. Emission at 700 nm was recorded using a filtre-equipped high-sensitivity (4-million-pixel) cooled charge-coupled device camera. Acquisition time was 30 s for each image, followed by a photography-style white light image or conventional X-ray for image fusion and co-registration of anatomical information.

For the imaging of Cy7-labelled compounds, excitation and emission wavelengths were 730 and 790 nm, respectively; acquisition time was 30 s.

For the time of examination, mice were held under isoflurane inhalation anaesthesia (2.5% isoflurane in air).

For each region of interest measured for imaging analysis, the mean fluorescence intensity (SI) and resulting standard deviation (s.d.) were determined.

From the acquired fluorescence signals of the target region (SI_target_), SNRs were calculated as SNR=SI_target_/s.d._background signal_.

If possible, the comparison of affected organs (target) and healthy organs (control) in the same animal CNRs were calculated as CNR=(SI_target_−SI_control_)/s.d._background_.

### PET and CT scanning

Animals were anaesthetized with isoflurane, and 10 MBq of ^18^F-FDG in 100 μl 0.9% saline was injected intravenously 1 h before each PET analysis. For PET acquisition, animals were placed on a heat-controlled multimodal scanning bed and PET list mode data were acquired for 15 min using the 32-module quadHIDAC scanner (Oxford Positron Systems), dedicated to small animal imaging. The scanner has an effective resolution of 0.7 mm (full-width at half-maximum) in the transaxial and axial directions when using an iterative resolution recovery reconstruction algorithm. Subsequently, the scanning bed was transferred to the computed tomography scanner (Inveon, Siemens Medical Solutions) and a medium resolution (25 μm) CT acquisition was performed for each mouse. PET data were reconstructed into a single image volume for each mouse with a voxel size of 0.4 × 0.4 × 0.4 mm^3^. CT was reconstructed into a volume data set with a voxel size of 0.007 × 0.007 × 0.007 mm^3^. Image data sets were co-registered using extrinsic markers attached to the multimodal scanning bed and commercially available image analysis software (Inveon Research Workplace, Siemens Medical Solutions).

### Immunohistochemistry

Immunohistochemistry of ear sections (cryo) or paw sections (paraffin) was performed as described earlier using purified rabbit anti-sera against murine S100A9 (refs 10, 13). Briefly, after inhibition of endogenous peroxidase activity in frozen tissue sections Fc receptors were blocked by incubating in PBS/1% BSA including 50% normal goat serum (NGS). Slides were immunostained in a two-step procedure of incubation of primary antibody or isotype control followed by a horseradish peroxidase-conjugated secondary antibody using AEC as chromogen. Images were acquired by using an upright microscope (Axioskop, Zeiss). Paws from arthritis experiments were fixed in 4% formaldehyde and joints were decalcified with 5% formic acid in PBS during 7 days. After dehydration and embedment in paraffin, sections of the paws were cut (7 μM) in a standardized manner and processed for S100A9 staining. Sections were treated with 1% H_2_O_2_ to inhibit endogenous peroxidase and 0.1% Triton/PBS for antigen retrieval and additionally incubated with rabbit anti-S100A9 antibodies followed by a goat-anti-rabbit biotinylated antibody and 3,3′ diaminobenzidine (DAB).

### Eliciting irritant and ACD

ICD was induced by the application of 1% croton oil in olive oil-acetone (1:4) to the dorsal surface of the right ear of mice (*n*=5 per group) for 24 h, whereas the left ear served as a control. FRI was performed at 24, 48, 72 and 96 h after tracer application, corresponding to 48, 72, 96 and 120 h after croton oil treatment, respectively. Subsequently, mice were killed and ears were snap-frozen in liquid nitrogen and transferred for immunohistochemistry.

To elicit ACD, C57BL/6 or Balb/c mice were sensitized by the application of 25 μl of 0.5% 2,4-dinitrofluoro-1-benzene (Sigma) in olive oil/acetone (1:4) to the shaved abdominal wall on two consecutive days. Six days later, mice were challenged with 15 μl of 0.4% 2,4-dinitrofluoro-1-benzene in olive oil/acetone on the dorsal surface of the right ear. At day 2, mice were injected with either a-S100A9-Cy5.5 or rabIgG-Cy5.5 or a combination of a-S100A9-Cy5.5/rabIgG-Cy7, a-S100A9-Cy5.5/a-S100A9-Cy7 or a-S100A9-Cy5.5/rabIgG-Cy5.5. FRI was performed at different time points after antibody application, as indicated in Fig. 2. The disease severity of ACD was monitored by measuring the ear swelling.

### Induction and imaging of CIA

Arthritis was induced in DBA/jdba1/j or C57BL/6 mice by immunization using bovine collagen type II as described in detail earlier^44^. Briefly, bovine collagen type II (bCII, MD Biosciences) was dissolved in 0.05 M acetic acid at a concentration of 2 mg ml^−1^. Mice were injected subcutaneously at the tail base with 100 μg bCII emulsified in Complete Freund’s Adjuvant (Difco). The animals were boosted at day 21 with an intraperitoneal injection of 100 μg bCII. The onset of polyarthritis occurred around 4–5 days later. Mice were regularly inspected from day 14 after disease induction and scored for swelling, erythema and deformation of each joint three times a week (CS0=no swelling, CS1=slight swelling and erythema, CS2=pronounced oedema including joint rigidity). Scoring of single joints was added up to a maximum possible score of eight per mouse (two per paw). Regarding the susceptibility of C57BL/6 mice, arthritis was evaluated using an extended scoring three-point scale: 0=normal; 0.5=erythema, light oedema; 1=mild but definitely visible, erythema and oedema of one digit/toe or limb; 2=erythema and moderate oedema of at least two digits/toe or limb; 3=erythema, severe oedema of the entire paw and/or rigidity. Imaging was performed after arthritis was clinically detectable in the majority of treated animals 24 h after tracer application of either a-S100A9-Cy5.5 or rabIgG-Cy5.5 or a combination of a-S100A9-Cy5.5/a-S100A8-Cy7. At the end of the experiment, mice were killed and serum was collected for S100A8/S100A9 quantification by ELISA. Hindpaws were excised and processed for histology.

### Experimental leishmaniasis

Cutaneous leishmaniasis was initiated in two different mouse strains (C57BL/6 and Balb/c) by the subcutaneous application of 2 × 10^7^ promastigotes (stationary phase) of *L. major* in 50 μl PBS into the right hind footpad. Footpad thickness of the infected in relation to the healthy foot was assessed for clinical monitoring of disease. FRI was performed at days 4 and 28 after infection (representative for an early and a late phase immune response), 24 h after tracer injection. In separate experiments, sera and footpad washouts were collected at days 4 or 28 after *L. major* inoculation for S100A8/S100A9 quantification by ELISA. Briefly, *L. major* infected and non-infected hindpaws were washed out by s.c. injection of 250 μl PBS. Without applying any force, in order to obtain secreted protein, approximately 250 μl of the draining liquid was collected and used for further analysis.

### Statistical analysis

Results are presented throughout as mean values±standard deviation (s.d.). *P*-values are given in the figure legends and values of *P*>0.05 were considered not to be significant. Statistical analyses were performed by parametric tests (*t*-test or one-way analysis of variance) and the Mann–Whitney *U*-test.

## Author contributions

Th.V. designed and supervised the study and experiments, performed animal studies and wrote the manuscript. M.E. designed the study and the experiments, performed optical imaging and wrote the manuscript. T.V., B.P., S.Z., K.R. and P.v.L. performed animal experiments and analysed the data. S.H. and A.F. performed PET and PET/CT experiments and analysed the data. C.G. and C.B. designed and supervised the optical imaging experiments and edited the manuscript. M.S. supervised the PET imaging experiments and edited the manuscript. J.R. designed the study and experiments and wrote the manuscript.

## Additional information

**How to cite this article:** Vogl, T. *et al.* Alarmin S100A8/S100A9 as a biomarker for molecular imaging of local inflammatory activity. *Nat. Commun.* 5:4593 doi: 10.1038/ncomms5593 (2014).

## Supplementary Material

Supplementary Information Supplementary Figures 1-3
