# Supplementary material for: Alarmin S100A8/S100A9 as a biomarker for molecular imaging of local inflammatory activity
Source: Nat Commun. 2014 Aug 6;5:4593. doi: 10.1038/ncomms5593 (PMC4143994; doi:10.1038/ncomms5593)
Supplement: Supplementary Information — Supplementary Figures 1-3 [file ncomms5593-s1.pdf]

Supplementary Figures

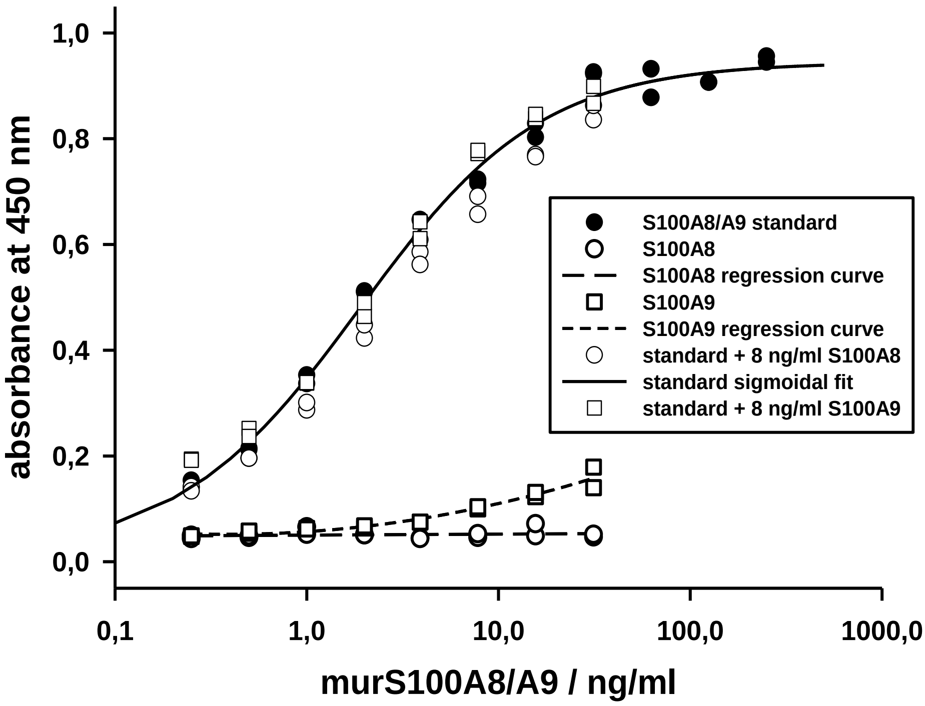

Supplementary Fig. 1: Specificity of the S100-ELISA for the S100A8/S100A9 heterodimer.

Wells were coated with the capturing polyclonal antibody anti-S100A8 (4 µg/ml, 50µl/well) and polyclonal anti-S100A9 (0.5 µg/ml, 50 µl/well), coupled to biotin according to the manufacturer's instructions, was used as a detection antibody. Streptavidin coupled HRP enzyme and TMB as a substrate was used for quantification by reading the absorbance at 405 nm using an MRX microplate reader (Dynex, Berlin). Purified recombinant heterodimer S100A8/S100A9 served as standard (filled circles; solid line: calculated sigmoidal standard curve). Neither S100A8 nor S100A9 homodimers alone (bold open circles and squares, dashed lines) were detected by the ELISA setting. The addition of neither S100A8 nor S100A9 homodimers to the heterodimer standard (open circles and squares) disturbed the results, indicating specificity for the S100A8/S100A9 heterodimer.

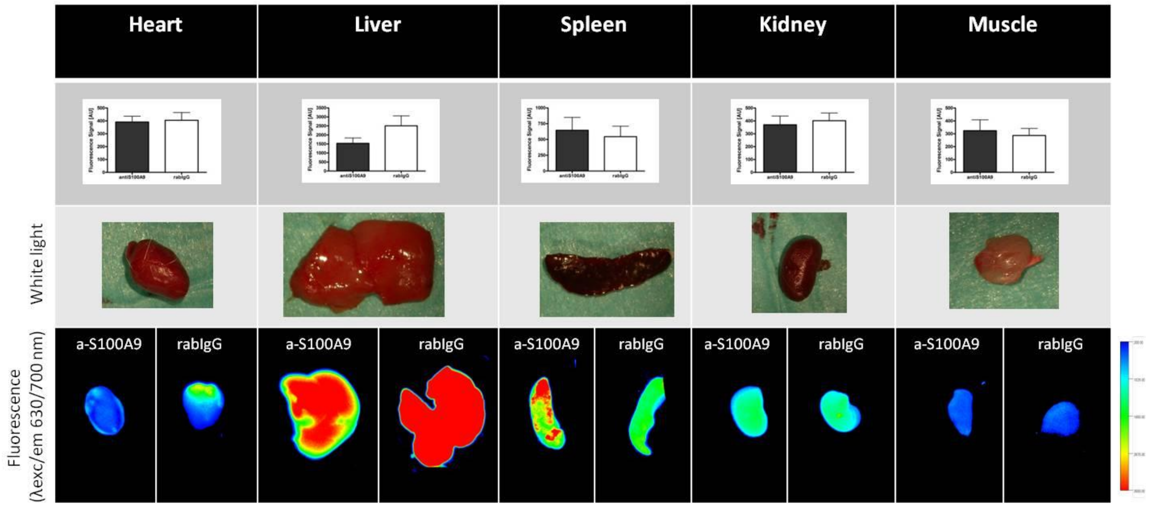

Supplementary Fig. 2: Biodistribution of injected Cy5.5 coupled antibodies.

Exemplary images of dissected organs of mice (n=5 for each group) sacrificed 24 h after injection of a-S100A9-Cy5.5 or rabIgG-Cy5.5 at a dose of 2 nmol equivalent Cy5.5/mouse. Graphs show the fluorescence of organs, reflecting dye accumulation. No significant differences between the distribution of a-S100A9-Cy5.5 and rabIgG-Cy5.5 could be detected in healthy animals. These findings support the suggestion that the measurable differences between diseased and healthy tissue as well as between the two probes in disease models indeed represent target specificity of the tracer.

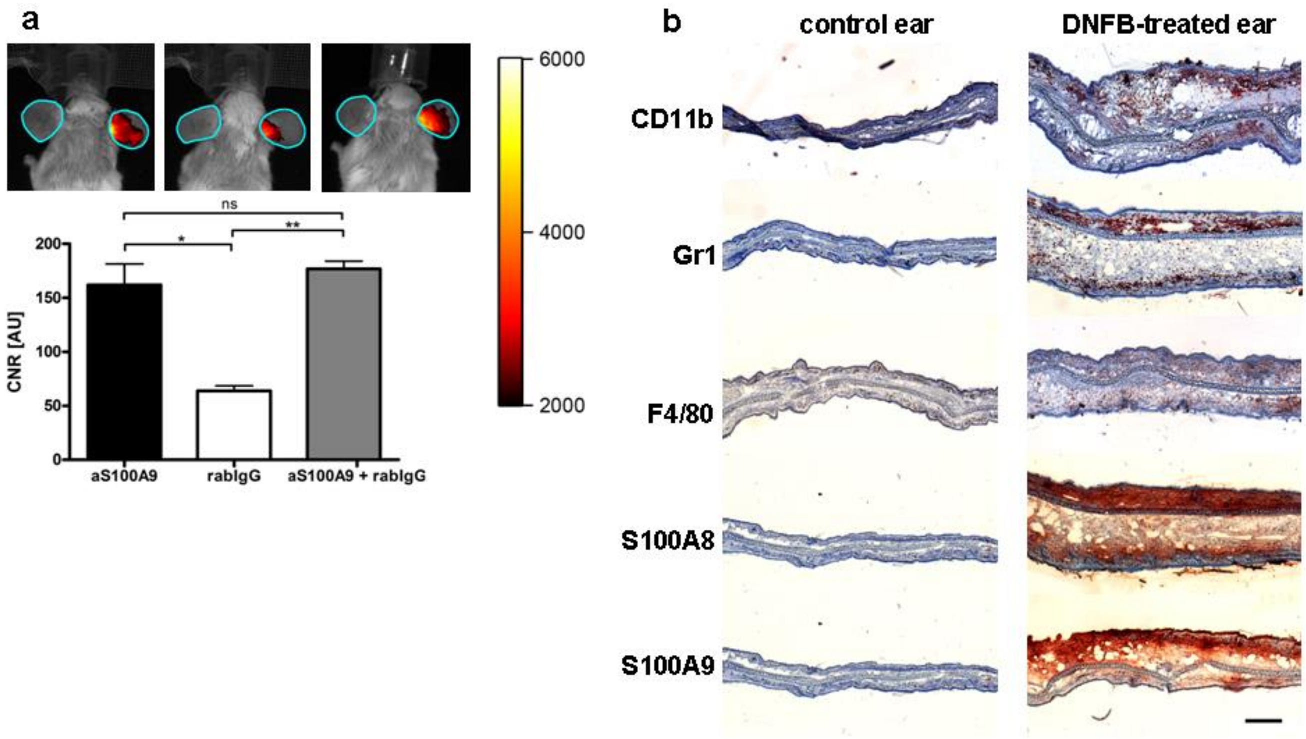

Supplementary Fig. 3: Simultaneous injection of a-S100A9-Cy5.5 and rabIgG-Cy5.5

(**a**) ACD was induced in mice and FRI was performed at 24 h after the application of either 2 nmol of a-S100A9-Cy5.5 or rabIgG-Cy5.5 or both antibodies (4 nmol in total) simultaneously. The comparison of CNR showed no significant changes in the affected ears after injection of either a-S100A9-Cy5.5 or both antibodies; both signals were significantly higher than the signal after application of rabIgG-Cy5.5 alone. Data are from 5 mice in each group (mean ± s.d., *p<0.05, **p<0.01, ns = not significant; t-test). AU = arbitrary units. (**b**) Histological analyses of ear sections from DNFB-treated mice 48 h after challenge (control ears = left sections, DNFB-treated ears = right sections. Sections were stained for immigrated immune cells (CD11b myeloid cells, Gr-1 granulocytes, F4/80 macrophages), S100A8 and S100A9. Scale bar, 250 µm.
